# PTSD SYMPTOMS AMONG VIETNAM, PERSIAN GULF, AND OEF/OIF/OND VETERANS: A RURAL/URBAN COMPARISON

**DOI:** 10.1093/geroni/igz038.1427

**Published:** 2019-11-08

**Authors:** Maria Kurth, Soyoung Choun, Dylan Lee, David Rothwell, Carolyn aldwin

**Affiliations:** 1 OSU Oregon State University, Corvallis, Oregon, United States; 2 Oregon State University, Corvallis, Oregon, United States

## Abstract

There are mixed results in studies examining rural/urban differences in PTSD symptoms among veterans; however, many of these studies failed to consider possible confounds with geographic location. This study examined rural/urban differences in PTSD symptoms by combat exposure, war cohort, and gender. The VALOR (Veterans Aging: Longitudinal studies in Oregon) pilot study sampled Vietnam, Persian Gulf, and OEF/OIF/OND war cohorts using an online survey. The sample (N=237, Mage=57.84, SD=12.68) was mainly male (65%), White (85%), and urban (75.95%); most reported combat exposure (71%). Participants completed measures of PTSD, combat exposure, and demographics. Results indicate no effect of cohort or rural/urban status on PTSD symptoms. There was a significant effect of combat exposure, F(1,224)=4.58, p=.03, and gender, F(1,224)=4.13, p =.04, with males reporting higher levels of PTSD symptoms and combat exposure. Contrary to our expectations, there were no effects of cohort or geographic location on PTSD symptoms.

